# Comprehensive analysis of smart bed comfort across varied resting conditions using quantitative measures

**DOI:** 10.1371/journal.pone.0327241

**Published:** 2025-07-03

**Authors:** Xiangtian Bai, Ming Zhong, Yonghong Liu, Yafan Hu, Jun Ma

**Affiliations:** 1 School of Design, Hunan University, Changsha, China; 2 Innovation Institute of Industrial Design and Machine Intelligence, Hunan University, Quanzhou, China; 3 Xiangya School of Nursing, Central South University, Changsha, China; Northwestern University Feinberg School of Medicine, UNITED STATES OF AMERICA

## Abstract

Smart beds have become increasingly accepted, and are concurrently reshaping their lifestyles. Addressing the limited ability of smart beds to cater to health requirements, this study investigated smart bed comfort across diverse typical conditions. Objective body pressure distribution and participant-reported perceived comfort were recorded during typical smart bed usage. Statistical analysis was utilized to investigate overall and local comfort variations across different conditions and the correlation between perceived comfort and body pressure distribution. Statistical analysis highlighted the importance of equalizing forces and minimizing peak pressures. Alongside mean pressure, peak pressure—particularly in the buttock, thigh, and shank areas—played a pivotal role in comfort evaluation. Optimal bed board partitioning and interlinked mechanisms between adjacent boards enhance body curve fit and overall comfort. Balancing body forces and preventing feelings of weightlessness significantly improve user comfort and health. This analysis has been used to develop a comfort prediction model for smart bed design.

## 1. Introduction

With the mounting pressure of modern life, an increasing number of individuals struggle to fall and stay asleep, leading to issues such as irritability or daytime fatigue [1]. Sleep duration and quality significantly impact overall health [2]. Worldwide, researchers have explored diverse fields to enhance sleep health, including bed and mattress design [3,4], cognitive behavioral therapy [5,6], exercise regimes [7], and health education [8]. Unlike pharmacological approaches, these methods are likely to produce sustained benefits devoid of risks or adverse effects. Alongside nocturnal sleep, brief naps are effective in improving concentration and sustaining vitality [9].

Recently, rapid advancements in smart technologies have marked a pivotal moment in the sleep health sector [10]. The global surge in technology and intelligence, particularly through IoT, cloud computing, and other AI, has swiftly propelled the sleep-related smart product industry towards diversification and ecological growth. The United States and Europe have witnessed peak development in global markets for smart beds and smart medical beds, both for healthcare facilities and homes, while the Asian market exhibits potential for substantial growth in the upcoming years [11]. Notably, smart beds employed at the 2022 Beijing Winter Olympics village garnered considerable attention and received unanimous praise from international athletes [12]. These innovations have been gradually integrated into China and other Asian countries, concurrently reshaping lifestyles. The bed comprises a structure and a software control system [13]. Regarding hardware structure, it consists of four parts: an adjustable bed board, bed frame, mattress, and soft cover. The software adjusts the bed board for an optimized sleeping position [14]. Quality comfortable smart beds protect the spine, relax muscles [14], and aid breathing [15]. Consequently, prioritizing ergonomic research in smart bed development is crucial.

Comfort research [16] for smart beds is vital. Prior studies have indicated that extended use of inappropriate smart beds can result in back pain, particularly in the lower back [17], significantly impacting the user experience and well-being. Sleep posture notably affects sleep quality and is especially crucial for conditions such as sleep apnoea [18] and pressure ulcers [19], extensively documented in studies [20–22]. Additionally, comfort encompasses not only physical aspects [23] but also individual subjective traits, such as sensibility, mentality, and experience, causing varied comfort in similar settings [24]. Researchers have combined physiological parameters with comfort evaluations to comprehend the pressure effects of resting items [25–28] and explore the correlation between physiology and perception. Although combining subjective and objective methods is common in resting product design, this approach is relatively scarce for smart beds.

Sleep habits in Asia contrast with those in Europe and North America [29]. Asians generally prefer supine sleep, leading to the design of smart beds catering to the Asian market, particularly emphasizing the supine posture [30]. In China, the residents of first-tier cities have progressively embraced softer beds [31]. However, sedentary individuals experiencing symptoms such as back and cervical pain often receive advice from the doctors to switch to a medium-firm mattress, resulting in subsequent symptom improvement [32], Some even use smart beds for rehabilitation and physiotherapy [33]. Considering diverse age and gender requirements for bed softness and support, disparities in perceived comfort exist [34,35]. Studies on bed-perceived comfort encompass intricate factors such as bed structure, mattress material, and user physiological traits. These factors and their interplay should be acknowledged and factored into smart-bed comfort assessment. Aligning with earlier sleep comfort studies [36,37], employing scales for comfort scoring emerges as pivotal.

In this study, we conducted experiments to access reliable body pressure distribution data from participants using a smart bed. Simultaneously, we collected subjective comfort scores and analyzed both subjective and objective indicators across multiple dimensions. Our goal was to provide more comprehensive and accurate empirical evidence for the design of smart bed and the improvement of the public’s sleep quality. Our investigation focused on the following key areas: variations in perceived comfort across different smart bed conditions, differences in body pressure distribution across different smart bed conditions, correlation analysis between body pressure distribution and perceived comfort, variation in body pressure distribution indicators across different Body Mass Index (BMI) [38,39] categories, and the effects of gender and lying time on local perceived comfort.

## 2. Materials and methods

### 2.1. Participants

This study was conducted to explore smart bed comfort across varied resting conditions using quantitative measures between March 2023 and April 2023 in Hunan province, China. A total of 40 participants (20 men and 20 women), including engineers, designers, and university students, were enrolled. The ranges, means, and standard deviations (SD) of the main anthropometric characteristics of the participants (age, weight, height, and BMI) are presented in Table 1.

**Table 1:** Main anthropometric characteristics of the participants.

	Men (20)			Women (20)		
	Range	Mean	SD	Range	Mean	SD
Age [year]	25–35	28.94	3.64	25–31	27.11	3.10
Weight [kg]	45–97.50	71.56	16.67	42–70	53.61	9.72
Height [cm]	161–190	174.13	7.47	152–168	160.89	5.37
BMI [kg/m^2^]	16.94–30.77	23.37	4.21	16.82–25.41	20.66	3.18

All participants had used a minimum of two types of smart health products including smart massage chairs, smart neck pillows, and smart treadmills. Moreover, each participant must use a smart health product within the last 2 months [40]. They reported being in good health and could participate in the experiment. Those with serious conditions such as backaches, musculoskeletal disorders, or those requiring medical follow-up were excluded. Finally, participants were advised to avoid strenuous exercise the day before the experiment.

Before the experiment, eligible participants were thoroughly briefed on the procedures, covering body pressure distribution measurement (BPDM) and subjective comfort evaluation (SCE). Written informed consent was obtained from all the participants. Additionally, participants completed a questionnaire regarding their attitudes toward smart health products and sleep health. Questionnaire responses indicated mixed feelings about smart health products, yet a positive view on sleep health.

### 2.2. Smart bed

**Fig 1:**
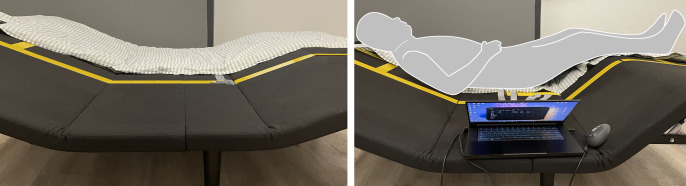
Smart bed with pressure sensor mat.

**Fig 2:**
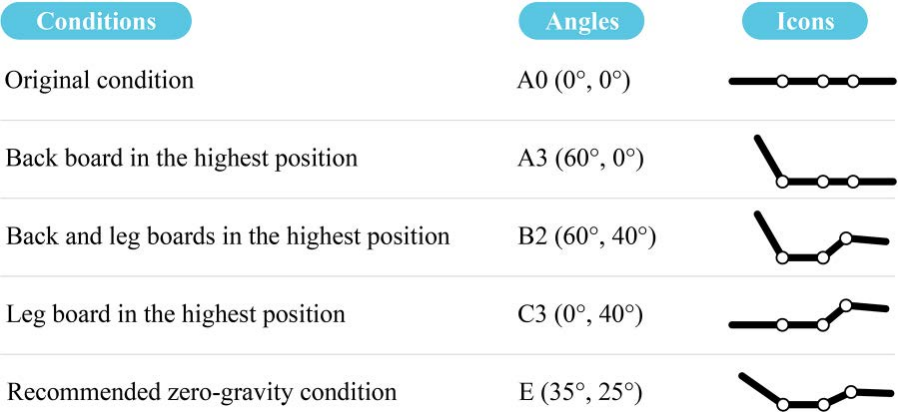
Four limit conditions and the zero-gravity condition of the smart bed adjustment.

The smart bed used in this comfort study features only bed board adjustments, omitting functions such as massage, sleep aid, and breathing monitoring to prevent added complexity (Fig 1). This basic smart bed serves as a prototype, effectively representing the primary design features. The prototype includes a stainless-steel bed frame and four adjustable bed boards linked by pivots. The bed boards were 180 cm long on one side and had varying lengths (65 cm, 50 cm, 32 cm, and 40 cm) on the other side, all with a thickness of 20 mm. With electric actuators, these bed boards pivot to adjust the smart bed to different conditions. The smart bed features four limit positions and a company-recommended zero-gravity condition, as presented in Fig 2. The zero-gravity condition was originally developed by NASA to minimize the pressure on astronauts during launch by evenly distributing gravitational forces and reducing pressure on joints and the spine [41,42]. Inspired by this principle, adjustable bed manufacturers discovered that this zero-gravity-like sensation could be achieved by adjusting the bed board during rest, which distributes body weight evenly and prevents excessive pressure on any one body part [43,44]. While there is some variation among manufacturers, the general goal of zero-gravity condition is to achieve a body-leg angle of approximately 120°, which positions the heart level with or slightly above the knees to minimize stress on the body and internal organs [43,45]. In the case of the smart bed used in this study, the preset angles for the backboard and legboard are 35° and 25°, respectively. Using this prototype, mattresses with varying thicknesses and support levels can be accommodated to meet diverse comfort needs. Soft covers, primarily leather, envelop the exposed structure.

### 2.3. Objective measurements

Throughout the smart bed testing, BPDM was measured by a pressure sensor – Legact Array Pressure Distribution RPPS-255*6 (Shenzhen, Guangdong, China) with a sampling rate of 10 Hz. The experimental pressure-sensing mat comprised 1530 sensing elements with a resolution of 30 mm. These measurements were supported by Windows MCD V1.5.0 software. A pressure sensor was placed at the center of the bed, where the participants rested, as shown in Fig 1. It recorded real-time pressure data for each sensor cell, and this data matrix was saved in Excel. For each test, software was employed to compute the overall pressure, mean pressure, contact area, peak pressure, and peak pressure point within each smart bed adjustment condition for every participant.

### 2.4. Subjective comfort

**Fig 3:**
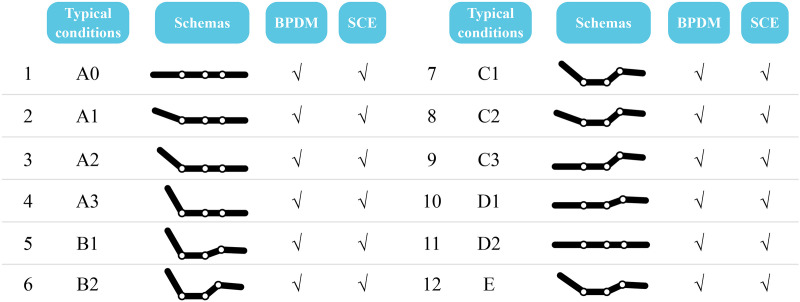
Typical smart bed conditions. * BPDM = body pressure distribution measurement; SCE = subjective comfort evaluation.

SCE was collected during the resting session by prompting participants to share their feelings about the resting experience. These SCEs were exclusively completed under typical smart bed conditions. These conditions include original condition (A0); back board at its highest position (A3); both back and leg boards at their highest position (B2); leg board at its highest (C3); every 20° rotation from A0 to A3 (A1 and A2); every 20° rotation from A3 to B2 (B1); every 20° rotation from B2 to C3 (C1 and C2); every 20° rotation from C3 to D2/A0 (D1) and “zero-gravity condition E” (see Fig 3).

**Fig 4:**
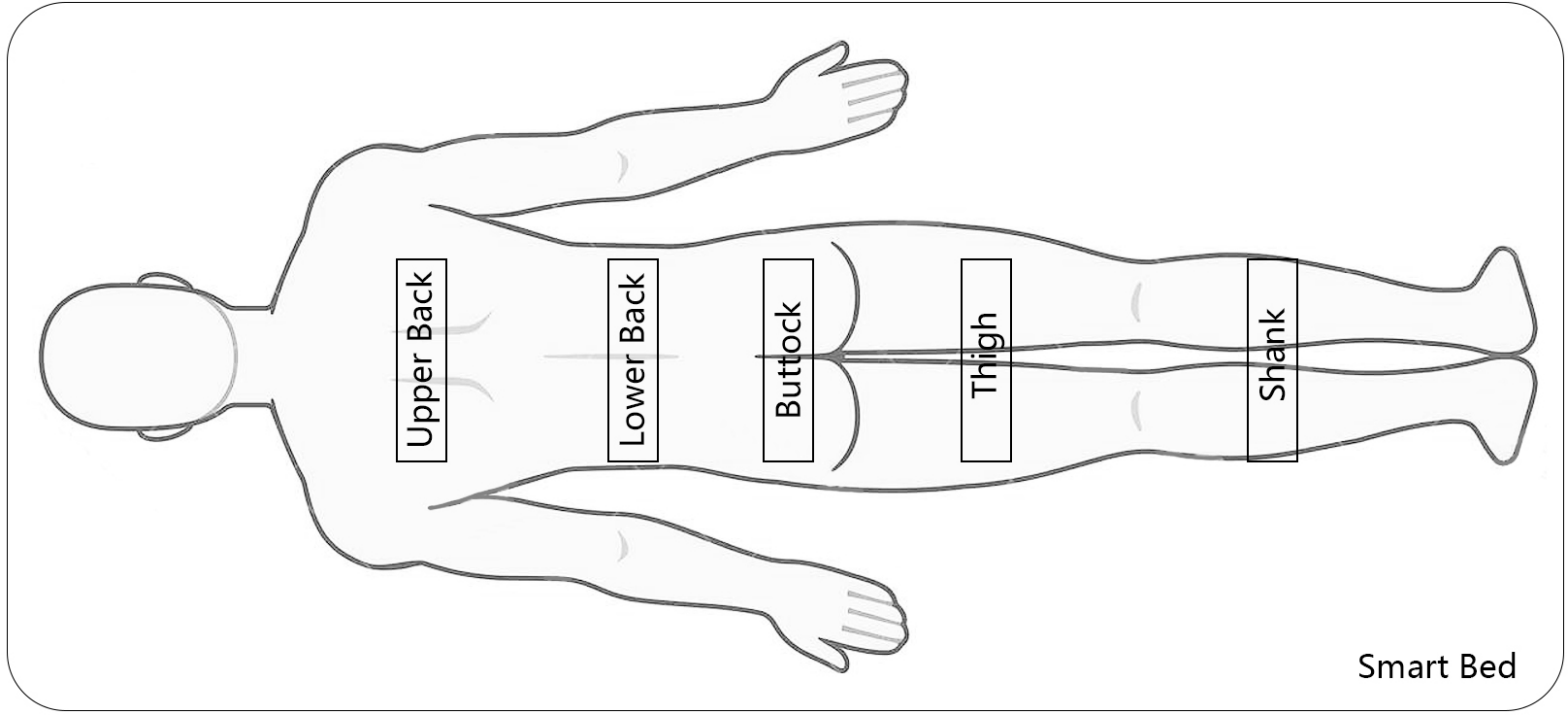
Contact area division between the body and smart bed.

Participants were tasked with evaluating their overall perceived comfort and local perceived comfort on a 7-point Likert scale [46], where ‘−3’ indicated least comfortable and ‘+3’ denoted most comfortable. This 7-point Likert scale strikes a suitable balance, allowing for sufficient discrimination in responses without an overwhelming number of options [47,48]. Considering the smart bed board arrangement, the body was broadly divided into four regions: upper back, lower back, buttock, thigh, and shank areas (Fig 4) [49]. Consequently, assessment items included: *‘Please assess the present overall comfort level of your body’, ‘Please assess the present comfort level of upper back area’, ‘Please assess the present comfort level of lower back area’, ‘Please assess the present comfort level of buttocks and thigh area’ and ‘Please assess the present comfort level of shank area’* (see Table 2).

**Table 2:** Subjective comfort evaluation questionnaire.

Perceived comfort	Rating						
Overall comfort	−3	−2	−1	0	1	2	3
	Extremely uncomfortable	Uncomfortable	Slightly uncomfortable	Uncertain	Slightly comfortable	Comfortable	Extremely comfortable
Upper back comfort	−3	−2	−1	0	1	2	3
	Extremely uncomfortable	Uncomfortable	Slightly uncomfortable	Uncertain	Slightly comfortable	Comfortable	Extremely comfortable
Lower back comfort	−3	−2	−1	0	1	2	3
	Extremely uncomfortable	Uncomfortable	Slightly uncomfortable	Uncertain	Slightly comfortable	Comfortable	Extremely comfortable
Buttocks and thigh comfort	−3	−2	−1	0	1	2	3
	Extremely uncomfortable	Uncomfortable	Slightly uncomfortable	Uncertain	Slightly comfortable	Comfortable	Extremely comfortable
Shank comfort	−3	−2	−1	0	1	2	3
	Extremely uncomfortable	Uncomfortable	Slightly uncomfortable	Uncertain	Slightly comfortable	Comfortable	Extremely comfortable

Another subjective evaluation was conducted both at the beginning and conclusion of the experiment to explore shifts in user interest in purchasing a smart bed. The same question was repeated the day after the experiment.

### 2.5. Experimental protocol

In the sleep laboratory setting, a mixed experimental design was employed to compare somatosensory shifts resulting from different smart bed adjustment conditions. We pre-generated 40 sets of non-repeating integer sequences ranging from 1 to 12. After the research assistant guided the participants into the laboratory, a set of sequences was randomly selected to determine their corresponding smart bed typical condition changes. In our pre-test practical study, we found that the majority of participants would frequently adjust the angles of the bed boards within a short period to achieve their preferred configuration before resting. We then consulted with experts on this phenomenon and, in combination with some sitting and lying product comfort studies, selected a 2-minute maintenance duration for each typical condition. Each user test lasted 24 min. The use of air conditioner and humidifier kept the experiment in a relatively stable environment (26 °C temperature and 40% humidity). Participants evaluated comfort in a fixed posture: buttocks at the middle bed board mark, head, back, and legs naturally extended in supine.

In the experiment, key independent variables were smart bed board adjustment and rotation, encompassing four limit conditions (see Fig 2). For each test run, body pressure distribution was consistently recorded, whereas SCE was collected under stable typical smart bed conditions, examining the impacts of operational mode configurations.

### 2.6. Statistical data analysis

Statistical analysis primarily addressed the following: (1) the relationship between overall body pressure distribution and bed condition changes, (2) body pressure distribution characteristics across varying BMI groups, (3) Variations in perceived comfort among different smart bed conditions, (4) disparities in local perceived comfort by gender and rest duration, and (5) the correlation between BPDM and SCE.

BPDM data, including overall pressure, contact area, and mean pressure, were directly acquired during testing (see Fig 3). Consider *k and l* as generic occupants, and let $P_{l,ij}^{k}$ represents the pressure over cell *ij* of the sensor matrix recorded at condition *l* by participant *k*. Correspondingly, $S_{l}^{k}\;$ is the contact area, where sensor matrix pressure is not zero. The mean pressure $\overset{―}{P_{l}^{k}}\;$ is ca*l*culated as follows:

$\overset{―}{P_{l}^{k}}=\sum_{i=1,j=1}^{I,J}\frac{P_{l,ij}^{k}}{S_{l}^{k}}$,

where *I*, *J* is the total number of sensor cells, where 1 ≤ *i *≤ 50, 1 ≤ *j *≤ 250.

Statistical analyses employed software (version 26.0; SPSS Inc., Chicago, IL, USA). Graphs depicted key objective data showcasing variations in overall pressure, mean pressure, contact area, peak pressure, and peak pressure point under diverse smart bed conditions. Moreover, discrepancies in objective data changes across distinct BMI groups were assessed. The Kruskal–Wallis test evaluated differences in perceived comfort among smart bed conditions, with Holm-Bonferroni-adjusted *p* values for pairwise comparisons. Spearman’s rank correlation coefficient explored the correlation between overall comfort and comfort within each smart bed condition. Mann–Whitney U test examined gender-based disparities in local perceived comfort. Wilcoxon Signed-Rank test analyzed the effects of resting duration on perceived comfort, assessing overall and local comfort scores collected in a fully lying posture (A0 and D2). The relationship between BPDM and SCE involved correlating mean and peak pressure values with perceived discomfort (see Fig 3).

All statistical tests were considered “significant” for *p*-value ≤ 0.05.

### 2.7. Ethics statement

This study was approved by the Ethics Review Committee, School of Design, Hunan University (E2022038). All methods were carried out in accordance with relevant guidelines and regulations. Written informed consent was obtained from all participants and their legal guardians.

## 3. Results

### 3.1. Variations in perceived comfort across different smart bed conditions

**Fig 5:**
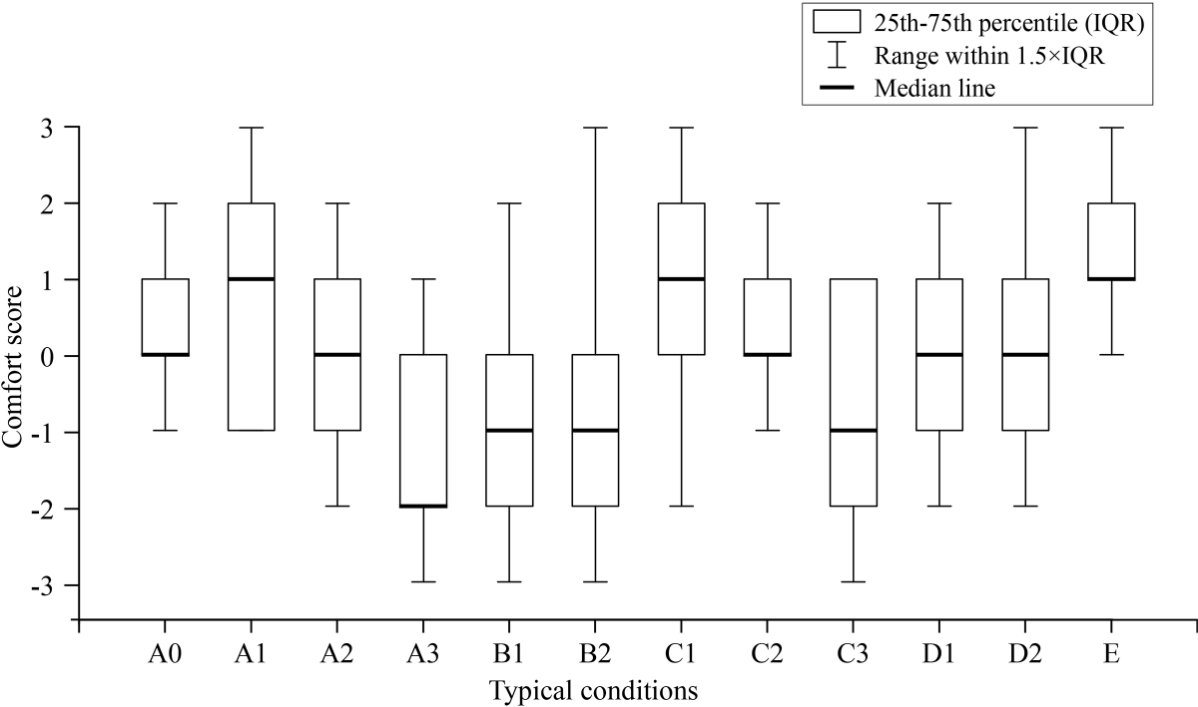
Mean changes in perceived comfort score among smart bed conditions.

As shown in Fig 5, the smart bed under condition A3 exhibited the lowest comfort score. Bivariate analysis showed a significant difference in perceived comfort among the smart bed conditions (H = 70.036, p = < 0.001) (Table 3). After applying the Holm-Bonferroni correction for multiple comparisons (p = 0.05), significant differences were found between A3 and A0, A1, C1, C2, E (all p < 0.05); between C3 and E, B1 and E, B2 and E, all p < 0.001, as shown in Table 4. Notably, several pairwise comparisons that were previously identified as significant (e.g., C3 versus C1; B1/B2 versus C1; A2 versus E) no longer reached significance after applying the more powerful Holm-Bonferroni procedure.

**Table 3:** Mean and Kruskal-Wallis test for perceived comfort among smart bed conditions.

	Median	Q1 (25th percentile)	Q3 (75th percentile)	H	*P* value
A0	0.00	0.00	1.00	70.036	< 0.001
A1	1.00	−1.00	2.00		
A2	0.00	−1.00	1.00		
A3	−2.00	−2.00	0.00		
B1	−1.00	−2.00	0.00		
B2	−1.00	−2.00	0.00		
C1	1.00	0.00	2.00		
C2	0.00	0.00	1.00		
C3	−1.00	−2.00	1.00		
D1	0.00	−1.00	1.00		
D2	0.00	−1.00	1.00		
E	1.00	1.00	2.00		

**Table 4:** Mean difference and significance of perceived comfort among smart bed conditions.

Condition (I)	Condition (J)	Mean Difference (I − J)	*P* value*	Holm-Bonferroni Adjusted *P*-value	Judgement
A3	A0	4.01	0.004	0.016	True
A3	C2	−4.21	0.002	0.010	True
A3	A1	4.52	< 0.001	0.002	True
A3	C1	−4.79	< 0.001	0.001	True
A3	E	−5.84	< 0.001	< 0.001	True
C3	C1	3.58	0.023	0.069	False
C3	E	−4.63	< 0.001	0.002	True
B1	C1	−3.55	0.026	0.078	False
B1	E	−4.59	< 0.001	0.003	True
B2	C1	−3.52	0.029	0.078	False
B2	E	−4.56	< 0.001	0.003	True
A2	E	−3.61	0.020	0.060	False

Note: *In this table, only statistically significant values are presented, i.e., *p* < 0.05.

Spearman’s rank correlation analysis revealed a positive correlation between the overall perceived comfort and local perceived comfort across all typical smart bed conditions (see Table 5). Compared with the other conditions, a stronger correlation (*r*_s_ > 0.80) exists between the overall comfort and upper-back area comfort in the back board lifting process (A0, A2, and A3).

**Table 5:** Spearman’s rank correlation (r_s_) between overall comfort and local comfort.

Area Comfort Statements	A0	A1	A2	A3	B1	B2	C1	C2	C3	D1	D2	E
The upper back area is comfortable.	0.822	0.774	0.803	0.815	0.605	0.620	0.791	0.661	0.595	0.788	0.704	0.691
The low back area is comfortable.	0.659	0.475		0.473	0.646	0.470	0.712	0.689	0.584	0.726	0.754	0.515
The buttocks and thigh area is comfortable.	0.510	0.649	0.693	0.439	0.500		0.651	0.687		0.516	0.685	0.619
The shank area is comfortable.	0.473		0.560	0.619			0.692	0.582		0.439	0.494	0.745

Note: In this table, only statistically significant values are presented, i.e., *p* < 0.05.

### 3.2. Differences in body pressure distribution across different smart bed conditions

**Fig 6:**
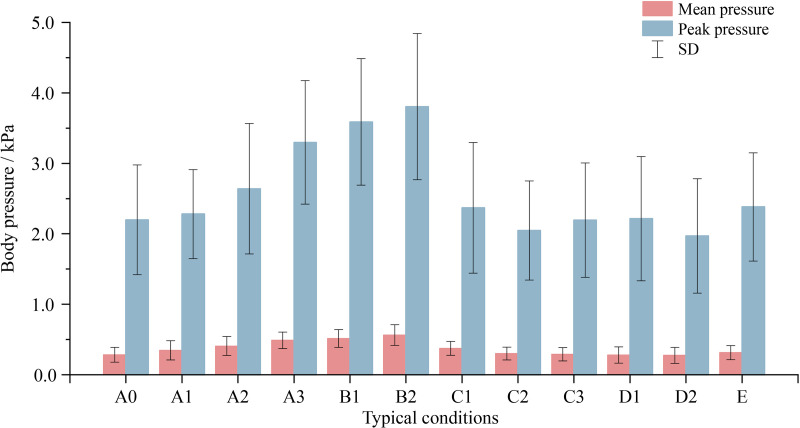
Relationship between mean pressure and peak pressure with changes in bed conditions.

**Fig 7:**
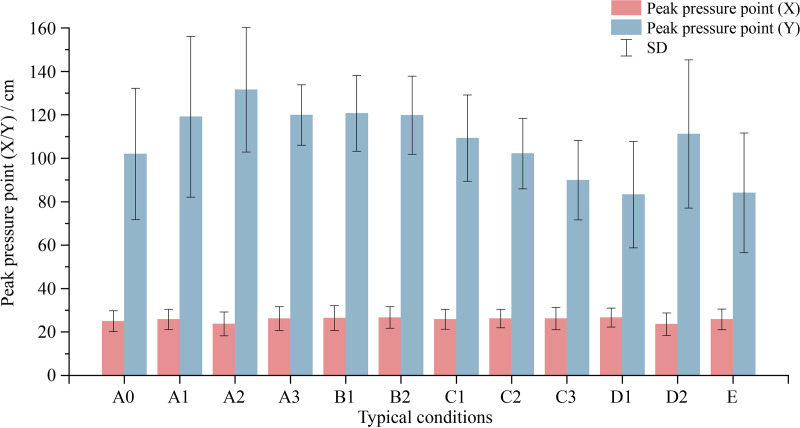
Quantitative analysis of peak pressure point across different bed conditions.

The key body pressure distribution indicators included mean pressure, peak pressure, and peak pressure point. The relationship between these indicators and changes in bed conditions is presented in Figs 6 and 7.

Generally, the trends in overall, mean and peak pressures were consistent. During supine positioning on the smart bed, pressures initially increased upon elevating the backboard. When the back and leg boards were at the highest position (B2), pressures reached their peak. Upon lowering the bed board, the indicators decreased, except for peak pressure, which experienced a slight rebound in conditions C3 and D1. However, the contact area was maximized when the back board was at the highest position and the leg board was not lifted (A3).

Regarding the peak pressure point, minimal variation was observed in the X-direction (perpendicular to the torso), while an overall trend of a brief downward shift followed by gradual upward movement in the Y-direction (parallel to the torso) was identified. With the lifting of the leg board (B1), the peak pressure point gradually ascended. As the backboard began to lower (C1), the upward movement rate of the peak pressure point intensified. Noteworthy downward displacement was observed until condition D2, where the peak pressure point coordinates diverged from the original setup (A0).

### 3.3. Correlation analysis between body pressure distribution and perceived comfort

As outlined in Table 6, different levels of correlation exist between the indicators included in the BPDM and SCE. Stronger correlations were found among the following variables: (1) comfort evaluations in the upper back area and overall pressure in the upper or lower back area; (2) comfort evaluations and mean pressure in the upper back area; (3) comfort evaluations and peak or mean pressure in the shank area; (4) peak pressure in the shank area and comfort evaluations in the lower back area; and (5) overall comfort and peak pressure in the buttocks and thigh area. A brief summary of other information showed that the overall pressure was mainly correlated with overall and localized comfort, particularly in the upper and lower back areas. Notably, peak pressure emerged as a pivotal factor affecting comfort evaluation. The peak pressure in the area below the buttocks was mainly correlated with overall and local comfort in the lower back or shank area.

**Table 6:** Spearman’s rank correlation (r_s_) between objective and subjective measures of comfort stratified by smart bed conditions.

Objective Measures	Subjective Measures	A0	A1	A2	A3	B1	B2	C1	C2	C3	D1	D2	E
Overall pressure over the smart bed	Upper back comfort											0.437	
	Lower back comfort		−0.458										
Overall pressure in the upper back area	Overall comfort		−0.401		−0.496								
	Upper back comfort	−0.600			−0.526								
Overall pressure in the lower back area	Overall comfort												−0.422
	Upper back comfort			0.512									
	Lower back comfort												−0.405
Overall pressure in the shank area	Lower back comfort			−0.410								−0.440	
	Shank comfort											−0.427	
Mean pressure over the smart bed	Overall comfort								0.401				
	Upper back comfort								0.403				
	Shank comfort												0.517
Mean pressure in the upper back area	Overall comfort				−0.431								
	Upper back comfort	−0.511			−0.598								
	Shank comfort	−0.463											
Mean pressure in the lower back area	Upper back comfort			0.431									
Mean pressure in the buttocks and thigh area	Upper back comfort								0.462				0.407
	Shank comfort												0.405
Mean pressure in the shank area	Overall comfort				−0.405								
	Lower back comfort			−0.478								−0.467	
	Shank comfort				−0.527							−0.407	
Peak pressure in the upper back area	Overall comfort				−0.474								
	Upper back comfort	−0.451			−0.457								
Peak pressure in the lower back area	Overall comfort										0.401		
	Upper back comfort										0.438		
	Lower back comfort			−0.484									
Peak pressure in the buttocks and thigh area	Overall comfort								0.508				
	Upper back comfort								0.440				
	Lower back comfort												0.481
Peak pressure in the shank area	Upper back comfort							−0.428					
	Lower back comfort			−0.560								−0.566	
	Buttocks and thigh comfort							−0.443					
	Shank comfort				−0.529				−0.530			−0.561	

Note: In this table, only statistically significant values are presented, i.e., *p* < 0.05.

### 3.4. Variation in body pressure distribution indicators across different Body Mass Index categories

**Fig 8:**
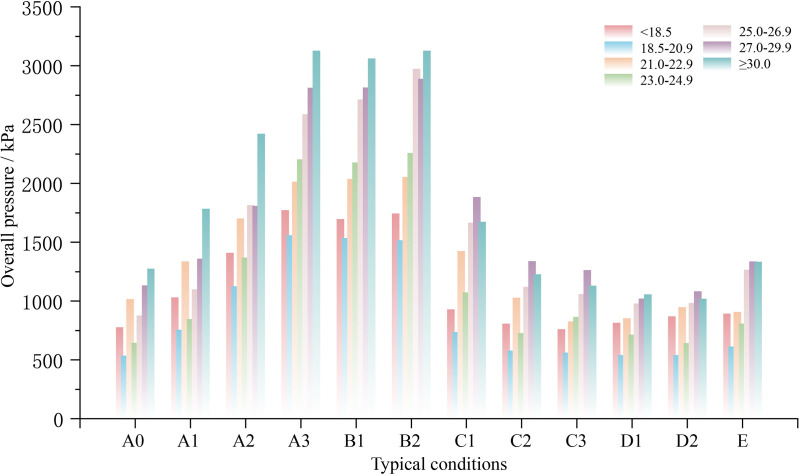
Characteristics of overall pressure across different BMI groups under varying bed conditions.

**Fig 9:**
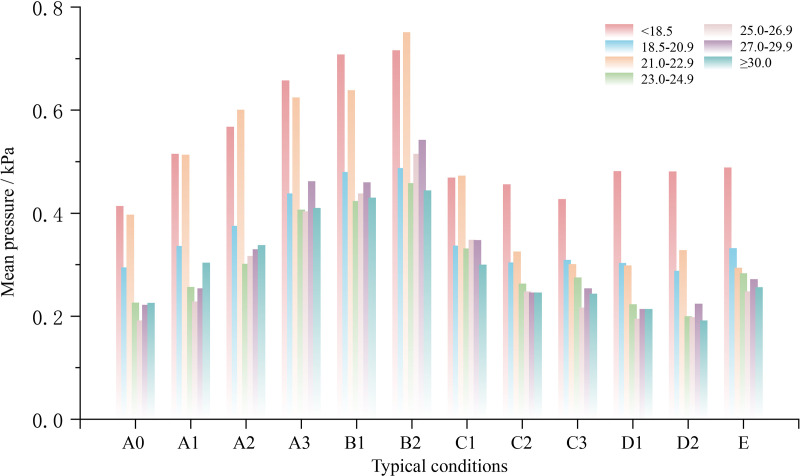
Characteristics of mean pressure across different BMI groups under varying bed conditions.

BMI serves as a global metric for body fat and overall health. By integrating participant BMI data (see Table 1) with BMI classification standards from various countries (the United States, China, and the UK), the Smart Bed BMI Classification employed in this study was defined as ‘<18.5’, ‘18.5–20.9’, ’21–22.9’, ’23–24.9’, ’25–26.9’, ’27–29.9’ and ‘≥30’. Consequently, the participant body pressure data were classified into seven groups. Overall pressure and mean pressure are illustrated as key indicators in Figs 8 and 9.

As shown in Fig 8, the trends in overall pressure remained generally consistent among the groups as the smart bed was adjusted. In most instances, groups with higher BMI values exhibited higher overall pressure. However, pressure values did not consistently exhibit a positive correlation with the BMI values, as observed in the ‘BMI < 18.5’ and ‘18.5 ≤ BMI ≤ 20.9’ groups. Here, the former consistently held a higher overall pressure than the latter, regardless of board condition changes. For mean pressure, the lower BMI groups (<18.5, 21.0–22.9) showed considerably higher mean pressure than the other groups. Additionally, the mean pressure values and trends were generally consistent in the other groups regardless of BMI.

### 3.5. The effects of gender and lying time on local perceived comfort

Mann–Whitney U test revealed no major differences between the genders in terms of local perceived comfort (see Table 7). The only exceptions were observed in the upper and lower back areas for conditions A3 and A2, respectively, specifically during the backboard lifting process. Notably, condition A0 represented the comfort level in the initial condition. Condition D2 represented the comfort level after 12 min of smart bed testing. Both conditions maintained identical bed settings. The Wilcoxon Signed-Rank test (A0–D2) showed a significant increase in perceived discomfort over time in the upper and lower back areas (Table 8). However, no significant change in the overall or local comfort was observed in the buttock, thigh, and shank areas.

**Table 7:** Mann–Whitney U test for local perceived comfort by gender. (*, *p* ≤ 0.05).

Area Comfort Statements	A0	A1	A2	A3	B1	B2	C1	C2	C3	D1	D2	E
The upper back area is comfortable.	0.136	0.934	0.207	0.032*	0.084	0.559	0.095	0.559	0.057	0.677	0.803	0.559
The low back area is comfortable.	0.677	0.452	0.023*	0.136	0.169	0.934	0.677	0.677	0.522	0.357	0.559	0.559
The buttocks and thigh area is comfortable.	0.978	0.846	0.637	0.978	0.677	0.718	0.419	0.487	0.598	0.276	0.207	0.760
The shank area is comfortable.	0.718	0.846	0.934	0.388	0.388	0.760	0.890	0.803	1.000	0.803	0.276	0.598

**Table 8:** Wilcoxon Signed-Rank test for comfort before and after smart bed conditions adjustment. (*, *p* ≤ 0.05).

	A0–D2	*P* value
Overall comfort	−1.387	0.166
Upper back comfort	−2.331*	0.020
Lower back comfort	−3.127*	0.002
Buttocks and thigh comfort	−1.328	0.184
Shank comfort	−1.724	0.085

## 4. Discussion

This study examined the impact of diverse smart bed conditions on body pressure distribution and perceived comfort during resting. Objective measures included overall pressure, contact area, mean pressure, peak pressure, and peak pressure point. Moreover, the relationship between SCE and BPDM was considered. This study aimed to comprehend how changes in smart bed conditions affect perceived comfort and body pressure distribution. This encompassed exploring the effects of factors such as gender and rest duration, on perceived comfort. Determining the correlations between objective and subjective variables can provide more comprehensive and accurate empirical evidence for the design of smart bed and the improvement of the public’s sleep quality.We tentatively determined that as the smart bed board was raised (A0 → A1 → A2 → A3 → B1 → B2), overall, mean, and peak pressures increased. This resulted in the users feeling uncomfortable, which aligns with previous research on other furniture products [50]. The correlation analysis between BPDM and SCE revealed that the overall pressure on the back area had the greatest influence on SCE, whereas the peak pressure in the lower body played a secondary role. Furthermore, higher pressure distribution was associated with greater user comfort. The smart bed used in the experiment comprised four linked bed boards to achieve the lifting operation, but this setup caused issues. The upper back area of the users aligned with just one bed board, increasing mattress material demand. This inadequacy fails to fulfill user comfort requirements. The recommendation is to further divide the bed boards and pair them with a coconut palm latex mattress. This combination would improve the fit to body shape and enhance overall smart bed comfort.

Specifically, when the smart bed back board was lifted (A0 → A1 → A2 → A3), a key reason for increased body pressure indicators was that the lifting back board folded with the buttocks board at an angle. This created extrusion pressure on the torso. This extrusion state was worsened by the continuous downward shift in the gravity center of the body [51,52]. Indirectly, this situation also resulted in the contact area being at its peak under condition A3. Uneven pressure distribution could raise all pressure indicators and significantly reduce comfort. Notably, the back board under condition A3 was lifted to 60°. As shown in the pressure nephogram, the extrusion pressure on the lower back reached its highest value during this, coinciding with increased contact area. The overall pressure across the smart bed and in the back area neared its peak, leading to relatively low comfort levels.

During the smart bed leg board lifting process (A3 → B1 → B2), although the overall pressure did not change significantly, raising the legs shifted the center of gravity upward. This effectively reduced the discomfort from back board extrusion pressure [53]. In terms of performance, the contact area was slightly reduced, mean pressure increased at a slower rate, and overall comfort substantially improved. Previous studies on body comfort have demonstrated that even pressure distribution and reduced extrusion sensation notably enhance user comfort [54,55]. To improve the overall comfort performance of smart bed lifting, designers and ergonomic researchers could focus on partitioning the bed boards within existing back board areas and refining the linkage mechanism between adjacent boards to prevent extrusion pressure.

As the smart bed back board began to lower (B2 → C1 → C2 → C3), all body pressure indicators rapidly decreased, whereas peak pressure point gradually moved upwards in the Y-direction. This decline was attributed to adequate support for the lower back, primarily causing this outcome [56]. When the leg board began to lower (C3 → D1 → D2), only the peak pressure notably changed with bed adjustments. However, conditions C3 and D1 corresponded to poorer comfort as the bed board lowered. Through in-depth interviews with the participants, poorer comfort performance was found to be related to a strong sense of weightlessness and body inversion. Some participants even reported extreme discomfort caused by increased head pressure.

Among the various conditions tested, higher comfort was observed when the smart bed was set to C1 and E conditions. This enhanced comfort was probably from comprehensive body support provided to areas such as the back, buttocks, and legs. Moreover, no pulling or extrusion sensations were experienced, and the overall forces were relatively balanced. The more the body experiences pulling or extrusion, the more force it has to actively exert, thereby becoming more uncomfortable. This supports the idea that the company-recommended zero-gravity condition (E) has scientific validity. Accordingly, the succeeding optimal smart bed design can be combined with a partitioned bed board for zero-gravity condition optimisation.

The participants were divided into seven groups based on their BMI values. Changes in body pressure indicators such as overall pressure, contact area, and mean pressure within smart bed conditions were observed in the different groups. First, the trends of these indicators were generally consistent across different BMI groups, suggesting that BMI can be used as a basis for smart bed products for various individuals. Smart beds can also utilize pressure data collected from a sensor matrix array to estimate user BMI values via a deep multitask neural network [57]. Second, mean pressure values and tendencies were generally consistent across most groups. This affirms that the mean pressure and its derivative indices are crucial for furniture product development and evaluation [38,58]. Notably, lower BMI groups (<18.5, 21.0–22.9) had higher mean pressure. A possible reason for this is that the lower BMI groups had less fat, and their bones made direct contact with the smart bed, which was insufficiently cushioned. A large gap between participant’s lower back in the lower BMI group and the bed board was observed, resulting in a small contact area and high mean pressure. Participants in higher BMI groups did not have such gaps in the back area. When designing for lower BMI individuals, increasing the number of backboard segments could offer partial back support. For gender-specific smart bed users, design considerations should focus on A2 and A3 conditions.

Perceived comfort performance showed significant differences among various smart bed conditions, particularly in condition A3, where comfort was notably lower, as seen in overall pressure and contact area indicators of BPDM. Conversely, C1 and E conditions, rated as more comfortable, differed significantly from A2, A3, B1, B2, and C3 conditions. These latter conditions shared two key features: (1) the back board lifting angle was up to a maximum of 60°, and (2) the difference in lifting angles between the back and leg boards exceeding 20°. Considering the body pressure data, the assumption is that the primary reason behind this lies in the instability of the body’s centre of gravity. The participants were required to actively exert force to relieve discomfort, which was consistent with objective measurements.

Statistical analysis of the local comfort perceptions found no significant differences in smart bed comfort perception between men and women. Only a few differences emerged in specific conditions (A2 and A3) in the back area. The reason why this result might be due to the change in the center of gravity. This finding contrasts with other studies on furniture comfort, where gender was considered an important factor in perceived comfort [34,59]. When comparing comfort data before and after the experiment, discomfort in the upper and lower back areas significantly increased over time. This underscores the importance of focusing on back board design and mattress selection of smart beds. In addition, the individual needs of the elderly or special populations (e.g., those with pressure ulcers, musculoskeletal disorders, or obstructive sleep apnea [60]), can affect their comfort level with smart beds. For instance, age-related declines in core strength among the elderly may increase comfort demands in specific body regions [61]. Similarly, pressure ulcers often occur over bony prominences, requiring strategies to minimize prolonged pressure in these areas.[62,63]. In the next study, we have recruited participants in the same proportion from the adolescent, young adult, middle-aged, and elderly groups, and plan to conduct body pressure and comfort studies with the same configurations across four age groups, comparing the effect of age on the comfort of the smart bed. At the same time, we took into account that prolonged use of smart beds does lead to excessive accumulation of pressure, which in turn brings about many pressure-related discomforts. In future studies, we will appropriately lengthen the testing time of participants in a single typical condition of the smart bed and conduct comparative studies in order to assess the changes in smart bed comfort.

Numerous studies have investigated the correlation between pressure variables and perceived comfort [24,28,64]. In our study, when examining the correlation between SCE and BPDM, we found that major body pressure indicators and local comfort were not entirely in alignment. Comfort in the upper back area is strongly correlated with overall pressure in the back area and mean pressure in the upper back area. This correlation is intuitively understandable. In well-designed smart beds, the transfer of partial pressure from the upper back area to the lower back or lumbar region is expected to improve comfort for the upper back and the entire body. Additionally, comfort in the shank area demonstrated a robust correlation with both peak and mean pressures in that region.

Similarly, experimental observations revealed that smart beds with lifting bed boards led to pressure concentration in the shank area, causing discomfort. Optimizing the bed surface structure or increasing the contact area could be considered as potential approaches to alleviate discomfort. The pressure indicators in different areas impact not just the comfort level of those regions but also contribute to the comfort of other areas to some extent. For example, a strong correlation exists between peak pressure in the shank area and lower back comfort, requiring further investigation into the mechanism. In summary, peak pressure, particularly in the buttock, thigh, and shank areas, is a critical factor in comfort evaluation. Therefore, optimizing the smart bed structure can achieve a more even pressure distribution throughout the body. This involves reducing the back-area load and peak pressures in the lower body area, effectively enhancing the smart bed comfort.

## 5. Conclusion

The proposed body comfort evaluation strategy (such as experimental design, materials and methods, and statistical data analysis) in this study yielded evidence for future research. This evidence can be used to develop a comfort prediction model for smart bed design, catering to long-term care for Asian populations. These results highlight that optimizing bed board partitioning and linkage patterns to align with the curve of the body enhances the overall comfort and health performance of the smart bed. Accordingly, maintaining the relative stability of the body’s center of gravity during smart bed adjustments is crucial. However, due to time and financial constraints, our sample size was limited and the characteristics of the included population may restrict the generalizability of our findings. We plan to conduct long-term comfort studies with a broader variation in subjects’ age, geography, education, and other dimensions to provide more comprehensive evidence. Moreover, participants provided assessments within constrained test conditions (fixed posture and activity). Finally, future research should investigate the relationship between smart bed comfort, shifts in free-lying positions, and movement activities. Future optimal smart bed designs may integrate subdivided bed boards for zero-gravity condition optimisation. This will help establish a comprehensive measurement system and indicator weights that impact smart bed comfort.

## Supporting information

S1 Data Raw data on objective body pressure distribution and participant-reported perceived comfort. (CSV)
